# Impacts of COVID-19 shelter in place across key life domains among immigrant farmworker Latina mothers and young adults

**DOI:** 10.1186/s12889-024-19438-1

**Published:** 2024-07-30

**Authors:** Michael Bakal, Elizabeth Ambriz, Lizbeth Ortiz-Pivaral, Katherine Kogut, Claire Snell Rood, Stephen Rauch, Brenda Eskenazi, Julianna Deardorff

**Affiliations:** 1 School of Public Health, University of California Berkeley, Berkeley, CA United States of America; 2 Department of Epidemiology & Biostatistics, University of California San Francisco, San Francisco, CA United States of America

**Keywords:** Stress, Hispanic/Latino, Mental Health, Farmworker, Qualitative research

## Abstract

**Objective:**

Individuals and families from racial and ethnic groups experience social and economic disadvantage making them vulnerable to the disproportionate impact of the COVID-19 pandemic. This study sought to capture the impacts of Shelter in Place (SIP) across key life domains including family life, education, work, mental health, and coping strategies among a sample of Mexican-origin mothers who were currently engaged in agricultural work, or whose spouses were engaged in agricultural work, and young adults who had a parent working in agriculture.

**Method:**

During the summer of 2020, while California was under SIP orders, we conducted three virtual focus groups using Zoom(r). We recruited focus group participants from the Center for the Health Assessment of Mothers and Children of Salinas (CHAMACOS), an ongoing, 20-year, longitudinal cohort study of Mexican-origin families in a predominantly agricultural area of California. Three focus groups were conducted with mothers (*n* = 9), mean age = 48 years, young adult women (*n* = 8) and young adult men (*n* = 5), mean age = 18 years, respectively.

**Results:**

Mothers reported high levels of stress stemming from fear of Covid-19 infection, work instability and financial concerns, children’s schooling, anxiety about an uncertain future, and the demands of caretaking for dependents. Adverse mental health impacts were particularly pronounced among participants experiencing multiple adversities pre-dating the pandemic, including unemployment, single motherhood, and having undocumented family members. For young adults, work instability and varying work hours were also a source of stress because they made it difficult to make decisions about the future, such as whether to attend college or how many classes to take. Families used coping strategies including expressing gratitude, focusing on what’s under one’s control, familismo, and community engagement to manage mental health challenges during SIP.

**Conclusion:**

In the event of future pandemics or disasters, particular attention is needed to those who experience unemployment, are undocumented and/or have undocumented family members, and/or are single parents facing economic adversity. During public health emergencies, action at the local, state, and national level is needed to support farmworkers and other vulnerable groups’ secondary major stressors stemming from inequities in access to affordable housing, childcare, living wages, healthcare, and other benefits.

**Supplementary Information:**

The online version contains supplementary material available at 10.1186/s12889-024-19438-1.

## Background

On March 13, 2020, during the first wave of the COVID-19 pandemic, the White House declared a state of national emergency in the United States. Shortly after, many states and counties began enacting shelter in place (SIP) orders intended to reduce human interaction and transmission of COVID-19 [1]. The impacts of the COVID-19 pandemic and SIP policies varied widely and disproportionately impacted Black, Indigenous, people of color (BIPOC), the low-income, and essential workers [2–6]. These marginalized groups experienced higher rates of COVID-19 infections, hospitalizations, and deaths [3, 4, 6]. BIPOC are overrepresented as “essential” or frontline workers who work in-person and were not able to SIP early in the COVID-19 crisis. These workers are often low paid, lack workplace protections such as employer-provided health care, appropriate sick leave, or paid time off, further increasing their vulnerability during a public health emergency [7, 8]. Furthermore, COVID-19 disparities also vary by occupation, with farmworkers who were also deemed “essential” amid the pandemic, and who are overwhelmingly Latino, disproportionately impacted by the earliest waves of COVID-19 [8–13]. 

### Farmworker health and living conditions pre-COVID-19

The approximately 3 million farmworkers in the US experience social and economic disadvantage and lack of access to conditions that support health [13]. Agriculture is one of the lowest paid occupations and among the most hazardous, with chronic exposure to substantial chemical and environmental toxicants [9]. In California, where this study is situated, farmworkers had an hourly mean wage of $14.40 and annual mean wage of $30,370 in May 2020 [14, 15]. In addition, farmworkers often live in substandard, unsafe, and overcrowded housing which has been linked to poor health outcomes [10, 11]. Furthermore, it is estimated that only one third of farmworker adults are insured [12, 13]. Lack of access to health insurance is a barrier preventing farmworkers from accessing health care. For example, the National Agricultural Workers Survey estimated that only 55.3% of farmworkers utilized US health care in the previous 2 years [13]. According the US Department of Agriculture (USDA), roughly half of farmworkers are undocumented [16]. Undocumented status among farmworkers has been associated with increased levels of stress, anxiety, depression, alcohol use, and poor mental health outcomes [17–19]. 

The COVID-19 pandemic and SIP orders produced high and enduring levels of psychosocial stress for individuals and families across the world, especially those who were already in a vulnerable position before the pandemic [20]. Studies have found consistent results about the adverse mental health impacts of COVID-19 and Shelter-in-Place, which included increased substance use and suicidal ideation [21, 22]. Given the directives for social distancing and isolation, families of essential workers—including farmworkers—were faced with several concerns, such as how to educate and care for their children at home while they were at work, and how to prevent disease transmission. These workers were engaged in tasks that put them at increased risk for virus contraction and were also anxious about transmitting the virus to family members [23, 24]. Essential workers experienced additional short- and long-term concerns during SIP; for instance, the implications of job loss, food and housing insecurity, and concerns about children’s learning and mental health [20]. A cross sectional study including Latino farmworkers (*n* = 1,115) between July 16 and November 30, 2020 in California found that the pandemic had exacerbated challenges affecting mental health and food security with nearly 20% of study participants reporting symptoms of depression and 15% reporting symptoms of anxiety. 6% reported an increase in their substance use and 37% experienced food insecurity during the pandemic [25]. 

A host of factors made farmworkers disproportionately vulnerable to the impact of COVID-19. These included overcrowded housing conditions [5, 9–11], riding to work with persons outside of their household, inadequate workplace training on COVID-19 transmission prevention, and inability to shelter-in-place during the peak of the pandemic [26]. A study on COVID-19 among farmworkers conducted by UC Berkeley researchers in collaboration with Clinica de Salud del Valle de Salinas found that many farmworkers were going to work while sick, many due to concerns about job or pay loss [27]. Among all adults tested for COVID-19 infection by clinics serving the Monterey County farmworker population, test positivity was 28% higher for farmworkers than for non-farmworkers from the same communities [28]. Another cross-sectional study of 1107 farmworkers in Monterey County, California found that both household and workplace risk factors, including living with children aged 5 years or younger or unrelated roommates and living or working with an individual with known or suspected COVID-19, were associated with positive results for COVID-19 infection [29]. Furthermore, a recent cross sectional study including 297 farmworkers from California found that 61.8%, a significant proportion of farmworkers experienced long COVID (defined as self-reported SARS-CoV-2 infection with symptoms > 28 days) with persistent symptoms that limit their ability to perform their work [30]. 

The current study used qualitative methods to address gaps in understanding by answering the research question about the impacts of SIP across key life domains including family life, education, work, mental health, and coping strategies among a sample of Mexican-origin mothers who were currently engaged in agricultural work, or whose spouses were engaged in agricultural work, and young adults who had a parent working in agriculture. A focus on women and in particular mothers is important because Latina women have longer life expectancy than Latino men, but more years in poorer health on average [31]. Furthermore, previous studies have found Latina women are likely to assist their children and others in their family in healthy lifestyle changes [32]. Findings from this study may inform responses to future public health crises by drawing attention to secondary psychosocial consequences that can emerge from a pandemic. Farmworkers remain disproportionately vulnerable to a range of public health threats, such as record heat and catastrophic wildfires stemming from climate change, future pandemics or cuts to the social safety net [33]. Understanding and responding not only to the crises themselves, but also the psychosocial challenges that accompany them, is critical.

## Methods

During the summer of 2020, while California was under SIP orders, we conducted three virtual focus groups using Zoom(r). We recruited participants from the Center for the Health Assessment of Mothers and Children of Salinas (CHAMACOS), anongoing longitudinal cohort study of Mexican-origin families in a predominantly agricultural area of California (total *n* = 600 families).Specifically, focus group participants were recruited from a subset of mothers (*n* = 273) and their young adult children (*n* = 198) who had completed a quantitative survey shortly after COVID-19 shelter-in-place restrictions were adopted in the area. The quantitative survey, which was administered by phone with mothers and independently online by young adults, included questions on demographics and household occupational status. It also included the USDA Food Security Scale [34], the General Anxiety Disorder 7-item scale (GAD-7) [35], the Center for Epidemiological Studies Depression 10-item scale (CES-D 10) [36, 37] for mothers, and the Behavior Assessment Scale for Children (BASC-2) [38] depression subscale in young adults. (Different depression scales were used to allow for direct comparison with previous data collected from these participants.) We used data from the quantitative survey to select focus group participants and to describe their characteristics relative to “non-participants” (i.e. people who completed the survey but did not participate in focus groups).

Of the participants who completed the survey, we used purposive sampling to recruit mothers who were currently engaged in agricultural work, or whose spouses were engaged in agricultural work. Young adult men and women were recruited if they had a parent working in agriculture. All focus group participants were recruited via phone.

We sent consent forms to those who expressed interest in participating via DocuSign(r), an electronic signature application. We scheduled individual meetings with CHAMACOS mothers to review the consent form and, if needed, engage in a practice session to illustrate how to use Zoom. Because young adults were familiar with the platform from online learning, no practice sessions were conducted with them. Each participant was compensated with a $75 debit card.

Three focus groups were conducted in total, one with mothers (*n* = 9), one with young adult women (*n* = 8) and one with young adult men (*n* = 5). Focus groups were conducted in Spanish (with mothers) and English (with young adult women and men) by two of the authors of the study (M.B. and L.O.P), who alternated between facilitation and note-taking roles. Focus groups followed a semi-structured protocol which sought to capture the impacts of SIP across key life domains including family, education, work, peer relationships, mental health, and coping strategies. Questions were developed for each domain and refined over the course of several team meetings in an iterative fashion based on the previously administered COVID-19 survey, the available literature on mental health impacts of the SIP order, and researchers’ familiarity with the study participants and context. Focus groups were approximately two hours in duration each.

We audio-recorded and transcribed verbatim (in the language that was spoken) all focus group discussions, and then conducted qualitative coding using Braun and Clark’s thematic analysis approach [39]. Given our interest in understanding SIP across key life domains, we began by deductively coding our data according to our broad domains of interest (family life, education, work, mental health, and coping strategies). Within each domain, we conducted inductive coding through a collaborative team process that included discussion andrepeated analysis of transcripts using with MAXQDA 2020 [40]. The authors (M.B. and L.O.P) reflected on their positionality in relation to the data and data analysis by keeping a memo throughout the research process. Additionally, M.B. and L.O.P engaged in member checking and consensus building efforts to ensure quality and trustworthiness of the data.

For example, within the family domain, we noticed that many mothers voiced concern over the possibility of a long-term family catastrophe, but then quickly explaining how they turned their attention to more immediate-term, actionable matters. Through multiple rounds of coding and discussion, we decided to code this as “focusing on what is under your control.” Once the team came to an agreement about final codes, the first (M.B) and third (L.O.P) authors conducted coding independently from one another, reviewed one another’s coded segments, came to consensus, and then wrote summaries of the meaning of each code, according to the process described by Braun and Clark [39].

## Results

### Demographic information

Table 1. displays demographic data for focus group participants and those in the CHAMACOS cohort who did not participate in focus groups; the groups were generally similar. The mean age for the 9 mothers who participated in focus groups was 48 years (range = 40–58), and all spoke Spanish as their primary language. Six had worked since the start of the pandemic. Six showed high or marginal food insecurity, and six lived at or below the poverty line. With regard to mental health, five participants scored above 10 on the CESD-10 depression scale. Two showed little to no anxiety; six showed mild anxiety, and 1 showed moderate anxiety.

The mean age for the thirteen young adults (8 women, 5 men) was 18 years (range = 18–19). Six had worked since the start of the pandemic. Ten reported high or marginal food insecurity. For mental health outcomes, two had clinically significant BASC scores for depression, and 2 scored “at risk.” The majority (7) scored in the average range for depressive symptoms. Most participants (7) showed little to no anxiety, while three showed moderate anxiety scores.

**Table 1:** CHAMACOS COVID focus group participants comparison between participants and non-participants

Parents			
	Focus Group Participants (*n* = 9)	Non-participants (*n* = 264)	p_diff^a^
	*n* (%) or M ± SD	*n* (%) or M ± SD	
Age category			
35–40	1 (11.1)	36 (13.7)	0.99
41–45	3 (33.3)	91 (34.6)	
46–50	3 (33.3)	80 (30.4)	
51–65	2 (22.2)	56 (21.3)	
Age (continuous)	48.0 ± 6.3	46.8 ± 5.3	0.50
Country of birth			
United States	0 (0.0)	22 (8.4)	0.61
Mexico	9 (100)	237 (90.1)	
Other	0 (0.0)	4 (1.5)	
Language			
Spanish	9 (100.0)	236 (89.4)	0.30
English	0 (0.0)	28 (10.6)	
Worked since COVID			
Worked	6 (66.7)	153 (58.0)	0.60
Did not work	3 (33.3)	111 (42.1)	
Housing density (persons/room)			
≤ 0.50	0 (0.0)	10 (5.5)	0.28
0.51-1.00	11 (84.6)	105 (57.4)	
1.01–1.50	1 (7.7)	40 (21.9)	
≥ 1.51	1 (7.7)	28 (5.3)	
Number of housemates	3.3 ± 2.4	4.5 ± 1.7	0.04
Health Insurance			
Has health insurance	6 (66.7)	175 (66.5)	0.79
Does not have health insurance	3 (33.3)	76 (28.9)	
Other/not sure	0 (0.0)	12 (4.6)	
Food security			
High or marginal	6 (66.7)	165 (62.5)	0.80
Low or very low	3 (33.3)	99 (37.5)	
Anxiety (GAD-7)			
Little to none	2 (22.2)	174 (65.9)	0.04
Mild	6 (66.7)	67 (25.4)	
Moderate	1 (11.1)	17 (6.4)	
Severe	0 (0.0)	6 (2.3)	
Anxiety (continuous)	5.9 ± 3.9	3.9 ± 4.1	0.15
Depression (CES-D)			
Depressed (≥ 10)	5 (55.6)	86 (32.6)	0.15
Not depressed (< 10)	4 (44.4)	178 (67.4)	
Depression (continuous)	8.9 ± 5.9	7.2 ± 6.5	0.44
Household Poverty (16-year visit)			
At or below poverty	6 (66.7)	147 (55.7)	0.51
Above poverty	3 (33.3)	117 (44.3)	

Young Adults			
	Focus Group Participants (*n* = 13)	Non-participants (*n* = 185)	p_diff^a^
	*n* (%) or M ± SD	*n* (%) or M ± SD	
Age			
18	4 (30.8)	48 (26.0)	0.62
19	9 (69.2)	125 (66.6)	
20	0 (0.0)	12 (6.5)	
Age (continuous)			
Worked since COVID			
Worked	6 (46.2)	94 (50.8)	0.75
Did not work	7 (53.9)	91 (49.2)	
Health Insurance			
Has health insurance	11 (84.6)	140 (75.7)	0.25
Does not have health insurance	2 (15.4)	17 (8.7)	
Other/not sure	0 (0.0)	29 (15.7)	
Food security			
High or marginal	10 (76.9)	147 (79.5)	0.83
Low or very low	3 (23.1)	38 (20.5)	
Number of housemates	4.2 ± 1.9	4.4 ± 1.7	0.78
Anxiety (GAD-7)			
Little to none	7 (53.9)	103 (56.3)	0.51
Mild	2 (15.4)	51 (27.9)	
Moderate	3 (23.1)	21 (11.5)	
Severe	1 (7.7)	8 (4.4)	
Anxiety (continuous)	6.0 (5.4)	4.9 (4.9)	0.43
Depression (BASC)			
Clinically significant	2 (15.4)	24 (13.0)	0.88
At risk	2 (15.4)	19 (10.3)	
Average	7 (53.9)	99 (53.5)	
Low	2 (15.4)	43 (23.2)	
Depression t-score (continuous)	51.7 ± 13.3	51.9 ± 12.8	0.95

^a^Chi-square test for categorical variables; t-test for continuous variables

### Educational impacts

Focus group participants commented that SIP restrictions had a dramatic impact on education for young adults, their younger siblings, and entire families. Prior to the COVID-19 pandemic, most of these young adults were enrolled in their first or second year of community college or university or were working to save money for college. For some of them, the onset of the pandemic led them to delay the start of college, citing fluctuating work hours, families’ financial precarity, and remote learning as key factors in their decision. One mother explained that her daughter:

*was planning on transferring to the 4 year university…one day she told me*, *‘You know what Mom I see that with COVID*, *with all that’s going on right now*, *a recession is going to hit and the cost of transferring to a university will be a big financial hit*, *so I’ve decided to [wait another year].*

However, delaying college was not seen in an entirely negative light. For example, some stated that delaying college enabled them to pick up additional work hours to support their families during a time of added financial burdens.

On the other hand, work uncertainty pushed two young adults in our focus group in the opposite direction: After their work hours were suddenly and drastically reduced, they used their free time to start college classes remotely. These young adults explained that the added flexibility of remote learning made to easier tostart taking college courses.

*I really didn’t have school on my agenda for at least another year or so*, *but seeing how things are going I’m probably gonna fast-track school and then probably start a little sooner than I expected.*

Almost all young adults in our focus groups reported a marked increase in theirfamilial responsibilities during SIP. The most time-intensive responsibility was helping younger siblings with homework. Although young adults generally reported taking on these responsibilities with a sense of dutifulness and even joy, they noted that at times the burdens felt overwhelming, with one saying,

*I take care of [my younger siblings]. I basically feel like I’m their mom and since I have classes now for Fall*, *[my parents] understand that I have classes*, *but at the same time they think of the younger siblings more because they sometimes tell me to take care of them when I have class and I’m not able to.*

Some young adults reported that their parents did not understand the kinds of time investments their education required, and felt that their parents often asked more of them than they could manage. Being at home, many reported, made it more difficult to concentrate on schoolwork, with one young adult male saying, “so I feel like if you’re in class [in person] you’re a bit more, like, disciplined…”.

Across all focus groups, mothers and young adults alike voiced serious concerns with the educational impacts that SIP would have on elementary-aged children. Two mothers had younger, elementary school-age children with special needs or who were learning English as a second language. These mothers expressed concern that their children were not receiving adequate academic support.Many lamented the lack of bilingual or dual-language programs available to support their emerging bilingual children. Among other problems, mothers explained that because homework assignments were given only in English, parents were unable to help their children. This was a source of frustration for mothers and meant that greater burdens fell on older siblings.

### Family life

With both young children and young adults taking classes online or living at home and working, SIP led nuclear families to spend far more time together. Most mothers said that they found this increase in family time to be a silver lining of the pandemic.

*And now*, *[with the pandemic] what happened? Well*, *my [young adult] son is going to take his classes virtually*, *so I have him here in the house; we eat together*, *have dinner together the way we did before and so I feel that we are united more as a family.*

On the other hand, many mothers experienced sadness at not being able to visit extended family members who they felt were important parts of their life. This included extended family members who lived nearby as well as those who lived abroad in Mexico.

Young adults reported both negative and positive aspects to the additional time spent with their nuclear families. Some reported feeling unproductive, saying that their home responsibilities were a distraction from their academics. Some said that they felt their parents did not understand their need for time to themselves to study and attend online classes, and some felt frustrated by lack of privacy. Those who moved away from home to attend college lamented the loss of new groups of friends they had begun to form. On the other hand, many reported being gratified to be able to help their parents with household chores and their younger siblings with schoolwork. Most young adults spoke about both the positive and negative aspects to SIP, as these quotations illustrate:

*So*, *I mean it’s on the positive side like when you’re getting closer as a family because it’s seven of us*, *but at the same time I get stuff put on [me] when I am not able to do it. So yeah*, *that’s on my privacy.*

*I think [SIP] brought out the good side and bad side out of [my family]. Some days it’s harder than others when you’re constantly in the same space as your siblings and your parents now.*

Young adults were also gratified by the opportunity to strengthen family relationships that were harder to maintain under pre-pandemic conditions. However, more time at home also meant greater proximity to relationships that were difficult or dysfunctional. For one young woman, pausing university studies due to SIP meant the end of the respite she enjoyed from what she perceived as her parents’ heavy-handed conservative values. Living under her parents’ roof again meant returning to difficult conflicts she had been grateful to leave behind when she went to college.

### Mental health

Mothers all reported high levels of stress stemming from fear of COVID-19 infection, work instability and financial concerns, children’s schooling, worrying about an uncertain future, and the demands of caretaking for dependents (both children and aging family members). Like their spouses, many of the mothers in our focus groups worked in the fields, in agricultural warehouses, or in other low-wage jobs where they experienced significant work instability. Some had recently lost their jobs or had their work hours significantly reduced. This caused stress to increase, as one mother said:

*Now that I don’t have work*, *I am very much affected; I have felt sick as a result of this.*

Many other mothers reported that the stress from SIP caused them to experience a variety of symptoms, including anxiety, difficulty sleeping, or headaches.

*I cannot sleep because of this*, *since “Oh my God*, *” if I become infected*, *what will happen to the kids and to my mom and to my children in college? It’s truly something very*, *very difficult for me. I am a single mother*, *and even the thought of getting sick [worries me]. I am the one who provides for my family*, *for my children.*

She suggested that what the number of family members who depend on her added to her stress, thus encapsulating a fear that many mothers expressed: If I were to die from COVID-19, who would take care of my family? This fear was particularly acute for single mothers, who often felt alone in bearing myriad responsibilities for keeping their families afloat. For those who worked in the fields or warehouses of the Salinas Valley, where cases of COVID-19 were among the highest in California, they faced a double-bind of needing to work to support their families, yet knowing that in doing so, they were potentially risking sickness or even death.

These various physical symptoms may illustrate the somatic manifestations of the chronic and acute stress that heads-of-household faced during the pandemic and shelter-in-place. Despite the near universal reports of stress among participants, adverse mental health impacts were particularly pronounced among those who experienced unemployment, who had undocumented family members, and/or who were single mothers. Moreover, many families experienced multiple adversities concurrently, which compounded the stress of the pandemic.

For young adults, work instability and varying work hours were also a source of stress. For example, one young man who worked as a security guard experienced drastic fluctuations in work hours, as SIP orders were instated, lifted, and then reinstated. Young adults reported that such changes to their work hours made it difficult to make decisions about the future, such as whether to attend college or university, or how many classes to take at a given time.

### Coping strategies

Mothers described an array of strategies they used to manage with stress. These included focusing on positive aspects of their lives, expressing gratitude, and focusing on what was under their control. For example, one mother said:

*You have to see that there is always a way out. The only thing that we can’t resolve is death.*

For many, these coping strategies were connected to religious and cultural practices — such as prayer, participating in church activities, and *familismo*, a Latino cultural value consisting of “strong family loyalty, closeness, and getting along with and contributing to the wellbeing of the nuclear family, extended family, and kinship networks” [41]. Familismo emerged as a key coping strategy among both women and young adults, who spoke frequently about time spent with family as part of what helped them cope with the stress of the pandemic.

Community service and activism also emerged as an important individual and community-level coping strategy. The start of the pandemic took place against the backdrop of harsh immigration rhetoric and policies implemented during the Trump Presidency, and farmworkers perceived their community to be especially vulnerable, having been denied access to the basic social protections afforded other groups. Many mothers expressed outrage at policies which only offered economic assistance to U.S. citizens. In response, several mothers began working on a mutual aid project which provided food and household items to the families of farmworkers who fell sick. A mother described participating in a “know your rights” campaign to assist undocumented immigrants and connect them with the resources they needed. Marking a contrast to political discourses that used citizenship status as the basis of inclusion/exclusion, one participant asserted, “*We are all brothers and sisters*.”

These coping strategies — expressing gratitude, focusing on what’s under one’s control, familismo, and community engagement — all helped families manage both the pragmatic and mental health challenges that the pandemic and SIP created.

## Discussion

In the coming years, farmworkers’ preexisting health issues may be exacerbated by future public health crises, such as record heat and catastrophic wildfires stemming from climate change, or pandemics [33]. It is critically important for public health professionals and policymakers to understand and prepare not only for the direct risks such crises pose to vulnerable groups, but also the secondary, psychosocial impacts such crises have on families and communities.

This paper addresses gaps in the literature about the impacts of SIP on farmworker families by examining the impacts of the COVID-19 pandemic and SIP orders on key life domains including family life, education, work, mental health, and coping strategies among Mexican origin families engaged in agricultural work. It also provides critical information about how to buffer the negative impacts and promote positive coping behaviors during future pandemics or public health emergencies among the most vulnerable.

Mothers and youth in farmworker families who participated in this study were situated differently compared to higher socioeconomic groups and non-essential higher paid occupations before the COVID-19 pandemic and experienced added vulnerability and impacts across life domains during SIP. Previous research demonstrates that the mental health burdens following community-wide disasters are extensive, with pervasive impacts on individuals and families, especially those who are the most vulnerable [42]. 

During the pandemic, farmworkers were not able to SIP because their work was considered an essential occupation– increasing their exposure to the virus and putting them at higher risk of infection. According to participants in the farmworker COVID-19 study, many farmworkers continued to go to work even when they were sick because they lacked essential workplace protections like paid sick leave, family leave, a living wage job, or were afraid to lose their jobs or wages [27, 43, 44]. In this study, all mothers reported high levels of stress stemming from fear of COVID-19 infection, work instability and financial precarity, and needing to work to support their families while knowing that they were risking sickness or even death. This stress was compounded by children’s schooling, specifically the lack of support for bilingual students and students with special needs, anxiety about an uncertain future, and the demands of caretaking for dependents. Many mothers, especially single mothers, expressed acute fear about dying during the pandemic and leaving their children alone without anyone to care for them. This is aligned with previous research that suggests that Latino cultural values such as familism and marianismo likely reinforce gender disparities in family caregiving [45, 46]. In this study the influence of gender roles seemed to play a role in caregiver burden with mothers demonstrating a high level of stress stemming caregiving responsibilities for their children during SIP.

For young adults, work instability and varying work hours were also a source of stress because it made it difficult to make decisions about the future, such as whether to attend college or University, or how many classes to take. Young adults in this study shared both positive and negative aspects of additional time spent with their nuclear families and mixed emotions about familismo. They reported the high level of responsibility they felt to support their families including taking care of their younger siblings and assisting them with schoolwork. For some young adults, more time at home also meant greater proximity to relationships that were difficult or dysfunctional. These findings are consistent with other studies have found that child and youth disaster outcomes are worst among children of highly distressed caregivers, or those caregivers who experience their own negative mental health outcomes from the disaster [20]. These findings align with the family stress model [47], which posits that economic hardship is associated with increased stress within the home, including conflict among family members [48, 49]. 

However, despite the compounded stressors these farmworker families experienced during SIP, they used coping strategies including expressing gratitude, focusing on what’s under one’s control, familismo, and community engagement to help themselves manage the mental health challenges during SIP. The women and young adults who participated in this study reported high levels of resilience and positive emotions, which have been found to buffer the negative effects of chronic stress and deal better with stress during times of major disruption and uncertainty [50]. Previous studies have found that higher family support related to better psychological well-being, whereas higher family conflict and lower support related to psychological distress in adults of Mexican origin [51]. Additional research suggests that familism exerts a protective influence among individuals coping with chronic stress [52]. One study found lower cortisol levels for adolescents with stronger alignment to Latino ethnic heritage values (e.g., familism, respect, religiosity), compared to relatively higher cortisol levels for those with less alignment to these values [53]. In addition, several mothers became more actively engaged in their community and began working on a mutual aid project, in which they provided food and household items to the families of farmworkers who fell sick. Some mothers participated in a “know your rights” campaign to assist undocumented immigrants and connect them with the resources they needed. In Monterey County, California, key community partnerships including clinicians, county government, agricultural industry representatives, and community members advocated for farmworker health and protections during the pandemic and included a call on the California government to provide masks to farmworkers [54]. Familism, expressing gratitude, and community engagement served as protective buffers during SIP, a time of major family and social disruption and uncertainty.

The impacts of COVID-19 SIP across key life domains including family life, education, work, mental health, and coping strategies among Mexican origin families engaged in agricultural work were exacerbated by the stark pre-existing inequities among farmworkers in Monterey County [27]. These essential workers lacked the essential workplace protections to protect themselves and their families during a public health emergency including having a living wage, paid sick leave, and family leave for this workforce [54]. The lack of economic stability and protections compounded the stressors experienced by farmworkers during SIP.

### Implications for policy and practice

To increase protections and decrease vulnerability during future public health emergencies, policy change at the local, state and federal government is necessary to ensure workers have the economic supports and workplace health and safety standards necessary to stay home if they fall ill with an infectious disease that could be spread in the workplace, and allow workers to stop working and self-isolate or care for a family member if they are sick. Ensuring economic supports by increasing access to access to affordable housing, rental assistance, childcare, food assistance, and living wages is essential since economic insecurity was a major stressor among participants, particularly among those who experienced unemployment, who had undocumented family members, and/or who were single mothers. Furthermore, support and parental engagement in the education system are needed for parents of bilingual students and students with special needs who felt inadequately prepared to support their children’s schooling during SIP. Additionally, a major stressor for young adults was having the responsibility of caring for younger siblings, this demonstrates the need to increase access to affordable childcare for farmworkers. Participants in this study used positive coping strategies during SIP, which could be used to inform interventions designed to buffer the negative impacts and promote positive coping behaviors during future pandemics or public health emergencies among the most vulnerable.

### Study limitations and challenges

This study includes a small sample size of mothers and young adults from the CHAMACOS cohort. In particular, only three focus groups were completed, one with each demographic group (mothers, young adult women and young adult men). Furthermore, although facilitating remote (Zoom) discussions were what was feasible and safest during SIP, virtual discussions could have been a barrier to participation for participants who did not have access to a computer or internet. However, findings from this study reveal the unique experience of farmworker mothers and young adults participating in the CHAMACOS cohort and in the specific context of Monterey County where the study was conducted. Furthermore, findings from this study can help offer insights and inform the development of a strategy to buffer the impacts of public health emergencies such as COVID-19 SIP on farmworkers, one of the most vulnerable occupations.

## Conclusion

Farmworker populations were disproportionately impacted by COVID-19 and SIP orders. Adverse mental health impacts were particularly pronounced among participants experiencing multiple adversities even before the pandemic. In the event of future pandemics or disasters, particular attention should be focused on those who experienced unemployment, had undocumented family members, and/or were single mothers. Action at the local, state, and national level is needed to engage vulnerable populations, including farmworkers, during public health emergencies in lifting industry workplace health and safety standards. Supports should address major stressors stemming from inequities in access to affordable housing, childcare, living wages, healthcare, and other benefits. Future studies should include men working in agriculture to determine if there are meaningful differences in the experiences of public health emergencies for women and men.

### Electronic supplementary material

Below is the link to the electronic supplementary material.

Supplementary Material 1

Supplementary Material 2

## Data Availability

No datasets were generated or analysed during the current study.

## Abbreviations

SIP: Shelter In Place
COVID-19: Coronavirus Disease 2019
CHAMACOS: Center for Health Assessment of Mothers and Children of Salinas Study

## References

[1] Berry CR, Fowler A, Glazer T, Handel-Meyer S, MacMillen A. Evaluating the effects of shelter-in-place policies during the COVID-19 pandemic. Proc Natl Acad Sci U S A. 2021;118(15):e2019706118. 10.1073/pnas.2019706118 33766888 10.1073/pnas.2019706118 PMC8053946

[2] Bambra C, Riordan R, Ford J, Matthews F. The COVID-19 pandemic and health inequalities. J Epidemiol Community Health. 2020;74(11):964–8. 10.1136/jech-2020-214401 32535550 10.1136/jech-2020-214401 PMC7298201

[3] Krieger N, ENOUGH. COVID-19, structural racism, police brutality, plutocracy, Climate Change-and Time for Health Justice, Democratic Governance, and an Equitable, sustainable future. Am J Public Health. 2020;110(11):1620–3. 10.2105/AJPH.2020.305886 32816556 10.2105/AJPH.2020.305886 PMC7542259

[4] Fortuna LR, Tolou-Shams M, Robles-Ramamurthy B, Porche MV. Inequity and the disproportionate impact of COVID-19 on communities of color in the United States: the need for a trauma-informed social justice response. Psychol Trauma. 2020;12(5):443–5. 10.1037/tra0000889 32478545 10.1037/tra0000889 PMC8243721

[5] Tai DBG, Shah A, Doubeni CA, Sia IG, Wieland ML. The disproportionate impact of COVID-19 on racial and ethnic minorities in the United States. Clin Infect Dis Published Online June. 2020;20. 10.1093/cid/ciaa815 10.1093/cid/ciaa815 PMC7337626 32562416

[6] Brown IM, Khan A, Slocum J, Campbell LF, Lacey JR, Landry AM. COVID-19 disparities and the Black Community: A Health Equity-Informed Rapid response is needed. Am J Public Health. 2020;110(9):1350–1. 10.2105/AJPH.2020.305804 32783709 10.2105/AJPH.2020.305804 PMC7427231

[7] Baker MG. Nonrelocatable occupations at increased risk during pandemics: United States, 2018. Am J Public Health. 2020;110(8):1126–32. 10.2105/AJPH.2020.305738 32552016 10.2105/AJPH.2020.305738 PMC7349441

[8] Dudley MJ. Reaching invisible and unprotected workers on farms during the Coronavirus Pandemic. J Agromedicine Published Online September. 2020;7:1–3. 10.1080/1059924X.2020.1815625 10.1080/1059924X.2020.1815625 32894685

[9] Summers P, Quandt SA, Talton JW, Galván L, Arcury TA. Hidden Farmworker Labor Camps in North Carolina: an Indicator of Structural Vulnerability. Am J Public Health. 2015;105(12):2570–5. 10.2105/AJPH.2015.302797 26469658 10.2105/AJPH.2015.302797 PMC4638263

[10] Marsh B, Milofsky C, Kissam E, Arcury TA. Understanding the role of Social Factors in Farmworker Housing and Health. New Solut. 2015;25(3):313–33. 10.1177/1048291115601020 26315036 10.1177/1048291115601020 PMC9107035

[11] Keim-Malpass J, Spears Johnson CR, Quandt SA, Arcury TA. Perceptions of housing conditions among migrant farmworkers and their families: implications for health, safety and social policy. Rural Remote Health. 2015;15:3076. 25682066 PMC4780055

[12] Services C. on CH. Health Care for Children of Farmworker Families. *Pediatrics*. 1995;95(6):952–953. 7761232

[13] Hoerster KD, Mayer JA, Gabbard S, et al. Impact of individual-, environmental-, and policy-level factors on health care utilization among us farmworkers. Am J Public Health. 2011;101(4):685–92. 10.2105/AJPH.2009.190892 21330594 10.2105/AJPH.2009.190892 PMC3052347

[14] Farmworkers, Laborers. Crop, Nursery, and Greenhouse. Accessed October 8, 2023. https://www.bls.gov/oes/2020/may/oes452092.htm

[15] The farmworker wage gap continued in 2020: Farmworkers and H-2A workers earned very low wages during the pandemic, even compared with other low-wage workers. Economic Policy Institute. Accessed October 8. 2023. https://www.epi.org/blog/the-farmworker-wage-gap-continued-in-2020-farmworkers-and-h-2a-workers-earned-very-low-wages-during-the-pandemic-even-compared-with-other-low-wage-workers/

[16] USDA ERS - Farm Labor. Accessed January 16. 2021. https://www.ers.usda.gov/topics/farm-economy/farm-labor/#legalstatus

[17] Cano MÁ, Sánchez M, Trepka MJ, et al. Immigration stress and Alcohol Use Severity among recently immigrated hispanic adults: examining moderating effects of gender, Immigration Status, and Social Support. J Clin Psychol. 2017;73(3):294–307. 10.1002/jclp.22330 27228112 10.1002/jclp.22330 PMC5159315

[18] Andrews IIIAR, Haws JK, Acosta LM, et al. Combinatorial effects of discrimination, legal status fears, adverse childhood experiences, and harsh working conditions among latino migrant farmworkers: testing learned helplessness hypotheses. J Latinx Psychol. 2020;8(3):179–201. 10.1037/lat0000141 10.1037/lat0000141 PMC7837582 33511335

[19] Hamilton ER, Hale JM, Savinar R. Immigrant legal status and health: legal status disparities in chronic conditions and Musculoskeletal Pain among Mexican-Born Farm workers in the United States. Demography. 2019;56(1):1–24. 10.1007/s13524-018-0746-8 30519846 10.1007/s13524-018-0746-8

[20] Liu CH, Doan SN. Psychosocial stress contagion in children and families during the COVID-19 pandemic. Clin Pediatr (Phila). 2020;59(9–10):853–5. 10.1177/0009922820927044 32462929 10.1177/0009922820927044

[21] Czeisler MÉ, Lane RI, Petrosky E, et al. Mental Health, Substance Use, and suicidal ideation during the COVID-19 pandemic - United States, June 24–30, 2020. MMWR Morb Mortal Wkly Rep. 2020;69(32):1049–57. 10.15585/mmwr.mm6932a1 32790653 10.15585/mmwr.mm6932a1 PMC7440121

[22] Galea S, Merchant RM, Lurie N. The Mental Health consequences of COVID-19 and physical distancing: the need for Prevention and early intervention. JAMA Intern Med. 2020;180(6):817–8. 10.1001/jamainternmed.2020.1562 32275292 10.1001/jamainternmed.2020.1562

[23] Haro-Ramos AY, Brown TT, Deardorff J, Aguilera A, Pollack Porter KM, Rodriguez HP. Frontline work and racial disparities in social and economic pandemic stressors during the first COVID-19 surge. Health Serv Res. 2023;58(Suppl 2):186–97. 10.1111/1475-6773.14136 36718961 10.1111/1475-6773.14136 PMC10339174

[24] Elahi NS, Abid G, Contreras F, Fernández IA. Work–family and family–work conflict and stress in times of COVID-19. Front Psychol. 2022;13:951149. 10.3389/fpsyg.2022.951149 36304883 10.3389/fpsyg.2022.951149 PMC9595337

[25] Mora AM, Lewnard JA, Rauch S, et al. Impact of COVID-19 pandemic on California farmworkers’ Mental Health and Food Security. J Agromedicine. 2022;27(3):303–14. 10.1080/1059924X.2022.2058664 35333134 10.1080/1059924X.2022.2058664

[26] FCCA. NATIONAL CENTER FOR FARMWORKER HEALTH, Accessed. October 18, 2023. https://www.ncfh.org/fcca.html

[27] December 2 KM. 2020December 4, 2020. California farmworkers hit hard by COVID-19, study finds. Berkeley News. Published December 2, 2020. Accessed December 6, 2020. https://news.berkeley.edu/2020/12/02/california-farmworkers-hit-hard-by-covid-19-study-finds/

[28] Lewnard JA, Mora AM, Nkwocha O, et al. Prevalence and Clinical Profile of severe Acute Respiratory Syndrome Coronavirus 2 infection among farmworkers, California, USA, June–November 2020. Emerg Infect Dis. 2021;27(5):1330–42. 10.3201/eid2705.204949 33657340 10.3201/eid2705.204949 PMC8084509

[29] Mora AM, Lewnard JA, Kogut K, et al. Risk factors Associated with SARS-CoV-2 infection among farmworkers in Monterey County, California. JAMA Netw Open. 2021;4(9):e2124116. 10.1001/jamanetworkopen.2021.24116 34524438 10.1001/jamanetworkopen.2021.24116 PMC8444020

[30] Mora AM, Kogut K, Sandhu NK, et al. SARS-CoV-2 infection and long COVID among California farmworkers. J Rural Health Published Online September. 2023;16. 10.1111/jrh.12796 10.1111/jrh.12796 37715721

[31] Bonow RO, Grant AO, Jacobs AK. The cardiovascular state of the union: confronting healthcare disparities. Circulation. 2005;111(10):1205–7. 10.1161/01.CIR.0000160705.97642.92 15769758 10.1161/01.CIR.0000160705.97642.92

[32] Paz K, Massey KP. Health disparity among Latina women: comparison with Non-latina Women. Clin Med Insights Womens Health. 2016;9(Suppl 1):71–4. 10.4137/CMWH.S38488 27478393 10.4137/CMWH.S38488 PMC4955974

[33] Brown P. FARMWORKER HEALTH IN CALIFORNIA.

[34] USDA ERS - Survey Tools. Accessed June 17. 2024. https://www.ers.usda.gov/topics/food-nutrition-assistance/food-security-in-the-u-s/survey-tools/#six

[35] Toussaint A, Hüsing P, Gumz A, et al. Sensitivity to change and minimal clinically important difference of the 7-item generalized anxiety disorder questionnaire (GAD-7). J Affect Disord. 2020;265:395–401. 10.1016/j.jad.2020.01.032 32090765 10.1016/j.jad.2020.01.032

[36] Andresen EM, Malmgren JA, Carter WB, Patrick DL. Screening for depression in well older adults: evaluation of a short form of the CES-D (center for epidemiologic studies Depression Scale). Am J Prev Med. 1994;10(2):77–84. 8037935 10.1016/S0749-3797(18)30622-6

[37] Lewinsohn PM, Seeley JR, Roberts RE, Allen NB. Center for epidemiologic studies Depression Scale (CES-D) as a screening instrument for depression among community-residing older adults. Psychol Aging. 1997;12(2):277–87. 10.1037/0882-7974.12.2.277 9189988 10.1037/0882-7974.12.2.277

[38] Reynolds CR. Behavior Assessment System for Children. The Corsini Encyclopedia of psychology. John Wiley & Sons, Ltd; 2010. pp. 1–2. 10.1002/9780470479216.corpsy0114

[39] Clarke V, Braun V. Thematic analysis. In: Teo T, editor. Encyclopedia of critical psychology. Springer; 2014. pp. 1947–52. 10.1007/978-1-4614-5583-7_311

[40] MAXQDA | All-In-One Qualitative & Mixed Methods Data Analysis Tool. MAXQDA. Accessed September 26. 2023. https://www.maxqda.com/

[41] Ayón C, Marsiglia FF, Bermudez-Parsai M, LATINO FAMILY MENTAL HEALTH:. EXPLORING THE ROLE OF DISCRIMINATION AND FAMILISMO. J Community Psychol. 2010;38(6):742–56. 10.1002/jcop.20392 20890371 10.1002/jcop.20392 PMC2947026

[42] Goyal D, Selix NW. Impact of COVID-19 on maternal Mental Health. MCN Am J Matern Child Nurs. 2021;46(2):103–9. 10.1097/NMC.0000000000000692 33470613 10.1097/NMC.0000000000000692 PMC7924923

[43] Ho V. Covid and California’s farmworkers: study lays bare disproportionate risks. *The Guardian*. https://www.theguardian.com/us-news/2020/dec/02/california-farmworkers-covid-19-infections. Published December 3, 2020. Accessed December 6, 2020.

[44] http://covid19farmworkerstudy.org/. Accessed December 6. 2020. http://covid19farmworkerstudy.org/

[45] Balbim GM, Magallanes M, Marques IG, et al. Sources of Caregiving Burden in Middle-aged and older latino caregivers. J Geriatr Psychiatry Neurol. 2020;33(4):185–94. 10.1177/0891988719874119 31510848 10.1177/0891988719874119

[46] Russell BS, Hutchison M, Tambling R, Tomkunas AJ, Horton AL. Initial challenges of Caregiving during COVID-19: Caregiver Burden, Mental Health, and the parent-child relationship. Child Psychiatry Hum Dev. 2020;51(5):671–82. 10.1007/s10578-020-01037-x 32749568 10.1007/s10578-020-01037-x PMC7398861

[47] Masarik AS, Conger RD. Stress and child development: a review of the family stress model. Curr Opin Psychol. 2017;13:85–90. 10.1016/j.copsyc.2016.05.008 28813301 10.1016/j.copsyc.2016.05.008

[48] Parra LA, O’Brien RP, Schrager SM, Goldbach JT. COVID-19-Related Household Job loss and Mental Health in a Nationwide United States sample of sexual minority adolescents. Behav Med. 2023;49(1):62–71. 10.1080/08964289.2021.1977604 34749595 10.1080/08964289.2021.1977604 PMC11453117

[49] Kelley HH, Lee Y, LeBaron-Black A, et al. Change in financial stress and relational wellbeing during COVID-19: exacerbating and alleviating influences. J Fam Econ Issues. 2023;44(1):34–52. 10.1007/s10834-022-09822-7 36820278 10.1007/s10834-022-09822-7 PMC9931994

[50] Chrisinger BW, Rich T, Lounsbury D, et al. Coping with the COVID-19 pandemic: contemplative practice behaviors are associated with better mental health outcomes and compliance with shelter-in-place orders in a prospective cohort study. Prev Med Rep. 2021;23:101451. 10.1016/j.pmedr.2021.101451 34189024 10.1016/j.pmedr.2021.101451 PMC8220389

[51] Rodriguez N, Mira CB, Paez ND, Myers HF. Exploring the complexities of familism and acculturation: central constructs for people of Mexican origin. Am J Community Psychol. 2007;39(1–2):61–77. 10.1007/s10464-007-9090-7 17437189 10.1007/s10464-007-9090-7

[52] Gallo LC, Penedo FJ, Espinosa de los Monteros K, Arguelles W. Resiliency in the Face of disadvantage: do hispanic cultural characteristics protect Health outcomes? J Pers. 2009;77(6):1707–46. 10.1111/j.1467-6494.2009.00598.x 19796063 10.1111/j.1467-6494.2009.00598.x

[53] Latino adolescents’ cultural. Values associated with diurnal cortisol activity. Psychoneuroendocrinology. 2019;109:104403. 10.1016/j.psyneuen.2019.104403 31437786 10.1016/j.psyneuen.2019.104403 PMC6842693

[54] Liebman AK, Seda CH, Galván AR. Farmworkers and COVID-19: community-based partnerships to address Health and Safety. Am J Public Health. 2021;111(8):1456–8. 10.2105/AJPH.2021.306323 34464192 10.2105/AJPH.2021.306323 PMC8489648

