# Supplementary material for: Impacts of COVID-19 shelter in place across key life domains among immigrant farmworker Latina mothers and young adults
Source: BMC Public Health. 2024 Jul 30;24:2036. doi: 10.1186/s12889-024-19438-1 (PMC11287914; doi:10.1186/s12889-024-19438-1)
Supplement: Supplementary file 1 — Supplementary Material 1 [file 12889_2024_19438_MOESM1_ESM.docx]

**PREGUNTAS PARA LAS MADRES:**

1. **[Apertura general: rutinas diarias]** Para empezar tómense un momento para reflexionar sobre sus rutinas diarias antes de la pandemia y ahora. ¿Cuáles diría que son los principales cambios a su rutina diaria antes de la pandemia y ahora? [**2:10-2:20]**
2. **[Impactos generales de la pandemia]** Ahora nos gustaría preguntarle sobre algunos de los cambios específicos que ha provocado la pandemia y cómo ha afectado a las personas de su familia y comunidad. **2:20-2:35**
   1. ¿Tuviste hijos en la escuela este año? ¿Conocías a personas con niños en la escuela? ¿Cómo fue la experiencia de las familias que tenían niños haciendo sus tareas escolares desde la casa?*
      1. Probe: ¿Qué desafíos han enfrentado las familias al ayudar a los niños con la tarea?*
      2. Probe: ¿Han surgido problemas relacionados con la tecnología u otros desafíos imprevistos con el trabajo escolar de los niños?
   2. Para las familias con niños, quisiera hablar acerca de lo que pasó después del año escolar: ¿cómo sienten que este verano es diferente a otros veranos?
   3. Con la cuarentena, las familias han pasado mucho más tiempo juntas. ¿Cómo crees que esto ha impactado a las familias, tanto positiva como negativamente?**
      1. Pregunte, si hay poca respuesta inicial: ¿Cómo ha afectado la pandemia la comunicación dentro de las familias, ya sea haciéndola más tensa y difícil o más fácil (por ejemplo, porque las personas están en casa más juntas)?
   4. ¿Cómo han adaptado las personas para cubrir los gastos domésticos?
      1. Probe: muchas personas en esta comunidad comparten sus ganancias con otros miembros de la familia, ya sea en los Estados Unidos o en el extranjero. ¿Cómo han cambiado estas demandas de remesas durante estos tiempos? ¿Cómo han equilibrado las familias estas demandas?
      2. Probe: ¿Creen que hay más familias sin suficiente para comer ahora que antes de la pandemia? Si es así, ¿sabe si han podido conseguir alimentos para comer en bancos de alimentos u otros lugares?
      3. Probe: ¿Las familias todavía han podido depender de los almuerzos escolares para las comidas de sus hijos? ¿Sabe si los almuerzos escolares todavía están disponibles durante los meses de verano?
3. [**Acceso a los servicios de salud]** Durante la pandemia, sabemos que las personas pueden necesitar atención médica, ya sea para hacerse pruebas o tratamiento para el coronavirus o para otros problemas de salud. **2:35-2:45**
   1. ¿Siente que las personas de su familia y amigos cercanos pueden hacerse la prueba del coronavirus si lo necesitan?**
      1. Probe: ¿Saben las personas de su comunidad qué hacer y adónde acudir si necesitan pruebas?**
      2. Probe: ¿Qué preocupaciones cree que podría tener la gente, especialmente los trabajadores agrícolas y sus familias, acerca del coronavirus y a la hora de querer hacerse una prueba?**
   2. ¿Qué pasa con el acceso a otros servicios de salud, no relacionados con el coronavirus? ¿Ha habido algún cambio en la frecuencia con la que las personas van a las citas médicas durante la pandemia comparado con antes de la pandemia?
4. [**Acceso a recursos de salud mental]** Sabemos que muchas personas estaban experimentando mucho estrés antes de la pandemia, y muchas están experimentando aún más estrés ahora ...**2:45-3:00**
   1. En general, ¿cómo cree que la pandemia ha afectado los niveles de estrés de las personas, especialmente las madres de familia?**
   2. ¿Cuáles son las principales preocupaciones que tienen las personas cercanas y familiares en este momento?
   3. ¿Cuáles son las formas en que las personas de su comunidad, especialmente las madres [madres de familia], enfrentan el estrés?
      1. Probe: ¿Las personas han buscado atención de salud mental en este momento o es muy raro?**
   4. Para las personas que han deseado atención de salud mental, les hace dificil encontrar ayuda?*
5. **[Condiciones laborales para los trabajadores agrícolas]** Durante la pandemia, los trabajadores agrícolas han sido considerados "trabajadores esenciales". ¿Qué ha significado eso en el contexto del trabajo diario? ¿Qué ha permanecido igual y qué ha cambiado para los trabajadores agrícolas desde el inicio de la pandemia?  **3:00-3:15**
   1. ¿Ha visto cambios en las horas de trabajo de los trabajadores agrícolas (aumentos o disminuciones? ¿Más o menos previsibilidad)?
   2. Que usted sepa, ¿qué se ha hecho para proporcionar a los trabajadores agrícolas información, recursos y equipo de protección (es decir, guantes, equipo de protección personal, mascarillas) y información sobre cómo prevenir el coronavirus?**
   3. ¿Qué tan adecuados cree que han sido estos esfuerzos? ¿Qué cambios adicionales piensan ustedes que se deben hacer, para proteger y apoyar mejor a los trabajadores agrícolas y sus familias?**
   4. ¿Cuál es su comprensión de las medidas que toman los trabajadores agrícolas si se enferman?**
      1. Probe: ¿Se quedan en casa? ¿Van a trabajar? ¿por qué?
      2. Probe: ¿Existe presión para trabajar de todos modos, incluso si están enfermos? Si es así, ¿de dónde viene esta presión (por ejemplo, de la propia necesidad económica de la familia / del jefe, del miedo a ser despedido, etc.) *
   5. ¿Qué tan accesibles son las pruebas de coronavirus para los trabajadores agrícolas? ¿Saben dónde hacerse la prueba si las necesitan?
6. **[Condiciones laborales para trabajadores no agrícolas]** Fuera del trabajo agrícola, sabemos que muchos otros están experimentando cambios en su trabajo. Para las personas que trabajan fuera de la agricultura, ¿cuáles son algunas de las formas en que ha cambiado el trabajo? **3:15-3:30**
   1. Aparte del trabajo agrícola, ¿cuáles son los otros tipos de trabajos más comunes que la gente de aquí de Salinas?
   2. ¿Ha visto algún cambio en las horas de trabajo (aumentos o disminuciones? Más o menos previsibilidad?
   3. ¿Qué se ha hecho para proporcionar a los trabajadores información, recursos y equipo de protección (es decir, guantes, equipo de protección personal, máscaras) e información sobre cómo prevenir el coronavirus?
   4. ¿Qué tan adecuados cree que han sido estos esfuerzos? ¿Qué cambios adicionales piensa usted que se deben realizar para proteger y apoyar mejor a los trabajadores y sus familias?
   5. ¿Cuáles son las medidas que toman los empleados si se enferman?
      1. Sonda: ¿Se quedan en casa? ¿Van a trabajar? ¿por qué?
      2. Probe: ¿Existe presión para trabajar de todos modos, incluso si está enfermo? Si es así, ¿de dónde viene la presión (por ejemplo, de la propia necesidad económica de la familia / del jefe, del miedo a ser despedido, etc.)
   6. ¿Qué tan accesibles son las pruebas de coronavirus para los empleados? ¿Saben dónde hacerse la prueba si lo necesitan?
7. [**Otros recursos]** ¿Qué otros recursos o servicios sociales cree que sus vecinos, familiares u otras personas en su comunidad han necesitado particularmente en este momento? ¿Ha podido la gente acceder a estos recursos? **3:30-3:35**

1. ¿Hay otras preocupaciones importantes que cree que son importantes plantear en este momento? **3:35-3:40**

| Roles M: Conectar, Escuchar; Preguntas de seguimiento: si aparecen nuevos términos o información (por ejemplo, referencias internas), haga la pregunta aclaratoria  L: lea la sala, alerta de cronometraje si surge una pregunta. Ambos nos presentamos.  El papel de L es tomar notas y también ayudar a asegurarnos de que estamos escuchando a todos; mantener el orden de los oradores; atenuar las preguntas a medida que avanzamos para mantener un registro del orden; enviar M texto si parece que estamos retrasados. |
| --- |
