# Supplementary material for: Impacts of COVID-19 shelter in place across key life domains among immigrant farmworker Latina mothers and young adults
Source: BMC Public Health. 2024 Jul 30;24:2036. doi: 10.1186/s12889-024-19438-1 (PMC11287914; doi:10.1186/s12889-024-19438-1)
Supplement: Supplementary file 2 — Supplementary Material 2 [file 12889_2024_19438_MOESM2_ESM.docx]

**QUESTIONS FOR YOUNG ADULTS**

1. **Lizbeth [General opening: daily routines] Take a moment to reflect on what your daily routines looked like before the pandemic, and now. What would you say are the main ways you feel that daily life has changed for you and others in your community?**
   1. **Probe: What have been the most challenging aspects of quarantine?**
   2. **With the quarantine, families have been spending much more time together. How do you think this has impacted families, both positively and negatively? ****
      1. **Probe: How have caretaking responsibilities been affected by the pandemic?****
      2. **Probe: (if there’s little initial response): Is there much of a difference as far as how you communicate and get along with your family?**
      3. **Probe: How, if at all, have young adults like yourselves been affected by younger siblings being home more?**
2. **Lizbeth [Peer Group Relationships] In light of shelter in place guidelines, how have you and your friends been impacted? So, a little more specifically, how have your friends and peers been affected by outside activities being disrupted or halted entirely?**
   1. **Probe: How have the ways you’ve stayed in touch with friends changed, if at all?****
      1. **Probe: Have young adults been getting together in person? If so, how often?**
      2. **Probe: To what extent have friends been able to stay in touch with each other? What do they do to stay in touch?**
      3. **Probe: Overall, has this situation changed friendships in any way?**
   2. **How do you think that your friends and peers feel about the measures to prevent spreading coronavirus, such as wearing masks and staying physically distant? Do you sense that people your age are following these guidelines? Why or why not?****
3. **[Mental Health] We know many people were experiencing lots of stress before the pandemic, and many are experiencing even more stress now…[Lizbeth]**
   1. **Overall, how do you feel that the pandemic has affected people’s stress levels? [Lizbeth]****
   2. **Michael What are the ways that you and your friends deal with stress? [Michael]**
      1. **Probe: Lots of people cope with stress by using alcohol, marijuana or other drugs. Do you think young people are using more or less during this time?****
      2. **Probe: Have people sought mental health care at this time or is that rare? ****
   3. **What are the main concerns that people in your community have at this time?**
      1. **Probe [if it comes up]: Have financial concerns been a bigger issue since the pandemic? Why or why not?**
   4. **We know that once people graduate from High School it’s harder to see a counselor. Do you see young people in your community struggling to get the help they need? ****

**---------------------Break--------------------------------------------------------------------------**

1. **[Accessing health services] During the pandemic, we know people may need to get medical care, either to get testing or treatment for coronavirus or for other health issues.**
   1. **Do you feel that young people in your community are able to get tested for coronavirus if they need it?**
      1. **Probe: Do young adults in your community know what to do and where to go if they need testing? ****
      2. **Probe: What concerns do you think people in your community might have around getting testing?****
      3. **Probe: For people in your community who are undocumented, do you sense that they are more or less scared about the risk of interacting with immigration officials than usual? ****
2. **[School & Work] Since the start of the pandemic, what have been the biggest changes to school/work activities? How have your friends and other people your age been adjusting to these changes?**
   1. **To probe schooling: How if at all, has the pandemic impacted schooling for people your age?****
      1. **Probe: Looking back to the Spring (when the pandemic first happened) how was schooling affected?**
      2. **Probe: what do their plans look like for the fall? Have plans changed at all?****
      3. **Probe: What college programs are people leaning toward? 2 year vs. 4 year college…**
      4. **Probe (if no responses in favor of 4 years): In what ways has COVID impacted people’s decision to pursue a 4-year degree, if at all?**
   2. **To probe work: How if at all, has the pandemic impacted young people’s jobs?****
      1. **To what extent have people taken on greater work responsibilities now and how has this affected other priorities?**
      2. **What concerns have people your age had about taking new jobs in light of COVID?**
      3. **Has it been harder to find jobs?**
   3. **To probe motivation: How if at all, has the pandemic impacted people’s motivation to stay in school and work towards a degree?****
      1. **Probe: What have you or other young adults you know done to stay motivated to work towards your goals?**
      2. **Probe: Has online learning had any impact on this? If so, what are the most challenging aspects of online learning?**
   4. **Overall how has the pandemic affected people's general outlook on this current stage of your life and the next few years of your life?**
3. **[Workplace safety] What kinds of jobs have people been able to work recently? What concerns do you feel young people have related to workplace safety? How do you feel these concerns are addressed?****
   1. **Do you know whether more of your friends and people your age have started doing agriculture work recently?****
      1. **Probe (If yes): To your knowledge, what has been done to provide farmworkers with information, resources, and protective equipment (i.e. glove, personal protective equipment, masks) on how to prevent coronavirus?****
   2. **What is your understanding of what measures workers take if they get sick?**
      1. **Probe: Do they stay home? Do they go to work? why?****
      2. **Probe: Is there pressure to work anyway, even if sick? If so, where does the pressure come from (e.g. from family’s own economic need/from the boss, from fear of being laid off, etc.) ****
4. **Are there any other major issues you’d like to mention at this time?**
